# Clinical, cytogenetic and molecular findings in nine Moroccan patients with Fanconi anemia

**DOI:** 10.11604/pamj.2021.39.72.27220

**Published:** 2021-05-26

**Authors:** Yassamine Doubaj, Abdelali Zrhidri, Siham Chafai Elalaoui, Jaber Lyahyai, Youssef El Kadiri, Nadia Elkassimi, Aziza Sbiti, Maria El Kababri, Laila Hessissen, Abdelaziz Sefiani

**Affiliations:** 1 Centre de Recherche en Génomique et Pathologies Humaines (Centre GENOPATH), Faculté de Médecine et de Pharmacie, Université Mohammed V, Rabat, Maroc,; 2 Département de Génétique Médicale, Institut National d'Hygiène, Rabat, Maroc,; 3 Centre d´Hématologie et Oncologie Pédiatrique, Hôpital d´Enfants, Rabat, Maroc

**Keywords:** Fanconi, anemia, cytogenetic, molecular, diagnosis, Moroccan

## Abstract

**Introduction:**

Fanconi anemia (FA) is a rare inherited hematological disease due to a defect in the DNA repair pathway resulting in congenital abnormalities and high susceptibility to develop cancers. The cytogenetic analysis using alkylating agents is still a reference test to establish the diagnosis. Despite the genetic heterogeneity, the identification of the causal mutation is actually performed especially after the development of next generation sequencing (NGS).

**Methods:**

we report here nine Moroccan patients referred to the department of Medical Genetics for suspicion of FA. We realized a genetic consultation to establish a clinical record with biological data before carrying out the genetic analysis. Karyotyping with mitomycin was performed for all the probands before elaborating molecular study. We used massively parallel sequencing to analyse the three most frequent mutated genes FANCA, FANCC, and FANCG, representing 84% of all genes involved in FA.

**Results:**

all the patients showed hematological signs associated with at least one extra-hematological congenital anomaly. The chromosomal breaks were significantly higher for the nine patients, compared to the controls. The molecular diagnosis was confirmed in 8 of the 9 families tested (88.8%) with 4 novel mutations. The next generation based sequencing identified 9 variations: 6 in the FANCA gene (66.6%), 3 in the FANCG gene (33.3%) and no FANCC variation was found. Of those, 7 were homozygous and 2 were compounds heterozygous.

**Conclusion:**

to the best of our knowledge, this is the first molecular report of Moroccan patients with FA suggesting the predominance of two genes without any recurrent mutation. The molecular analysis of FANCA and FANCG genes should be offered first for all patients in Morocco.

## Introduction

Fanconi anemia (FA) is a rare hematological disease clinically and genetically heterogeneous belonging to inherited bone marrow failure syndromes. Its prevalence is estimated to 3/1,000,000 [1]. In some populations, there is a higher frequency due to a founder effect. The frequency of heterozygous subjects is 1/181 in North America and 1/93 in Israel [2]. Besides an early onset of anemia in childhood, patients may present extra-hematological physical signs and have an increased risk of malignancies (acute myeloid leukemias and solid tumors). The FA cells have hypersensitivity to alkylating agents due to a defect in deoxyribonucleic acid (DNA) repair genes. The diagnosis is established by cytogenetic analysis of lymphocytes with Diepoxybutane (DEB) or mitomycin C (MMC), revealing a higher rate of chromosome breakage with radial forms comparing to blood normal controls. According to FA mutation database, 22 genes are known actually to cause FA with three heritability patterns, thus the molecular study was hard until the development of new technologies in sequencing allowing the rapid analysis of mutations and large rearrangement [3]. Hereby, we describe nine patients referred to the Department of Medical Genetics for suspicion of FA. Karyotyping with mitomycin C was performed for all the probands before elaborating molecular study using next generation sequencing. The purpose of the current study was to define the molecular spectrum of Fanconi Anemia in Moroccan patients by characterizing the genes and the mutations most often involved in Moroccan population in order to implement low-cost genetic tests for patients.

## Methods

**Study population:** nine children were addressed to the Department of Medical Genetics in Rabat for suspected FA. Patients were seen during a genetic consultation to collect all clinical and biological data. The parental reproductive history was recorded and a three generations pedigree was done for every family. An informed parental consent was obtained before the implementation of the genetic study reported here.

### Laboratory analysis

**Cytogenetic analysis:** carried out on peripheral blood sample in a heparinized tube, the study included lymphocyte culture for 72 hours at 37°C in a fully supplemented cell culture medium (Gibco, PB-MAX Karyotyping medium). The age and sex-matched control cultures were set up at the same time. The Mitomycin C (36ng/ml) was added 24 hours before harvesting metaphases. After stopping the cell cycle by adding karyomax colcemid solution, a hypotonic KCl (0.075 M) treatment was realized, followed by fixation using methanol and acetic acid fixative [4]. The chromosomal preparations obtained were spread on slides on hot plate and then stained with the Giemsa stain. At least 50 metaphases were scored for the chromosomal breaks such as chromatid breaks, dicentric and acentric chromosome, double minute, tri and tetra radials figures. Acentric and double minute chromosomes were counted as two breaks.

**Molecular analysis:** genomic DNA was extracted from peripheral blood using Invitrogen Kit (PureLink™ Quick Gel Extraction and polymerase chain reaction (PCR) Purification Combo Kit/K220001), and dosed by Qubit dsDNA HS (high sensitivity). A customized panel was designed online using on demand in Ion ampliSeq designer 6.0.6. We included in this panel 22 genes involved in different diseases from our consultation, eg: CFTR gene of cystic fibrosis, ATP7B gene of Wilson syndrome, GBA gene of Gaucher disease, SRCAP gene of Floating-Harbor syndrome. For FA, three genes, FANCA, FANCC and FANCG, known to be 84% causatives of FA were selected for targeted sequencing. The final customized panel was composed of average 562 amplicons, divided into two primer pools and in silico covered 100% of regions of interest (ROI). Libraries were prepared using Ion AmpliSeq Library Kit v2.0 (Life technologies, Carlsbad, CA, USA), according to the manufacturer´s instructions. One of 16 barcodes of the Ion Xpress Barcode Adapters1-16 Kit (Thermo fisher scientific life sciences solutions, Carlsbad, CA, USA) was added to each sample. Libraries were quantified with QubitdsDNA HS Assay Kit on Qubit 2.0 Fluorometer (molecular probes, eugene, OR, USA) and equimolar amounts of each library were used to prepare template for clonal amplification. Emulsion PCR with Ion PGM HI-Q OT2 Kit (Life technologies, Carlsbad, CA, USA) was performed on OneTouch2 Systems (Life technologies, Carlsbad, CA, USA). Templates were enriched using Ion OneTouch ES (Life technologies, Carlsbad, CA, USA) and prepared for 316v2 chip loading (Life technologies, Carlsbad, CA, USA). Sequencing runs were performed on Ion Torrent personal genome machine (PGM, Life Technologies) using Ion PGM HI-Q Sequencing, according to the manufacturer´s instructions. Generated raw sequence data in FASTQ format were aligned to the hg19 human reference genome using the Torrent mapping alignment program aligner implemented in v5.4 of the torrent suite software (Thermo fisher scientific). For SNV calling, we used plug-in Torrent Variant Caller v5.2.0.34 (Thermo fisher scientific) to generate a variant call format file. For torrent variant caller analysis, default setting of germline low-stringency parameters (minimal variant frequency of 0.1, minimum variant quality of 10, minimum coverage of 5x, maximum strand bias of 0.98, and minimum variant score of 10) were used and candidate variants were obtained only when variant frequency at a given position of ≥ 20% and variant coverage of ≥ 20x. Reported variants were confirmed in the human gene mutation database (HGMD), clinvar and previous publications. Amino acid predictions were performed using the SIFT algorithm, Mutationtaster and PolyPhen2 software tools.

**Ethical considerations:** all patients or their legal representatives gave written informed consent to the study, which was performed in accordance with the declaration of Helsinki protocols and approved by the local institutional review boards.

## Results

**Clinical presentation:** in our cohort, there were 6 girls and 3 boys. Their ages ranged from 4 to 11 years with a median age of 5 years. The consanguinity was present in 5 families (55%), the parents of patient 2 were originated from the same region. There was no family history of similar cases except for one patient who had three paternal cousins with hematological manifestations. Clinically, 77% of the patients had short stature, the typical facial features (triangular, micrognathia and mid-face hypoplasia) and the skeletal abnormalities were the most frequent physical signs with a frequency of 55%. The skin pigmentation anomalies were observed in 44% of cases (Table 1). At the hemogram, three patients had pancytopenia (33.3%), the six reminding patients had anemia associated with thrombocytopenia (44.4%) or neutropenia (22% patients). The bone marrow biopsy was realized for five patients revealing medullar hypoplasia for all of them except one who had bone marrow aplasia.

**Table 1:** clinical and biological characteristics of the FA patients

Patients	Age (yrs)	Sex	Parental consanguinity	SS	FF	SKA	SP	Others	Hemogram	Marrow biopsy
**P1**	5	F	+	+	-	-	-	Renal agenesis	Pancytopenia	Medullar aplasia
**P2**	6	F	-	+	+	Hypoplastic thumb, scoliosis	-	Ano-rectal malformation, left ectopic kidney	Anemia+ thrombocytopenia	NA
**P3**	5	F	+	+	+	-	+	-	Anemia+ thrombocytopenia	Medullar hypoplasia
**P4**	11	F	-	+	-	Polydactylia ; radio-cubital synostosis	+	-	Anemia+ neutropenia	NA
**P5**	5	F	+	+	-	Polydactylia	-	-	Anemia+ neutropenia	Medullar hypoplasia
**P6**	8	M	-	-	-	-	+	Hypospadias	Pancytopenia	Medullar hypoplasia
**P7**	4	F	+	+	+	Proximally placed thumb	-	-	Anemia+ thrombocytopenia	NA
**P8**	7	M	+	+	+	-	-	-	Anemia+ thrombocytopenia	NA
**P9**	4,5	M	+	-	+	Hypoplastic right thumb	+	-	Pancytopenia	Medullar hypoplasia

SS : short stature, FF : facial features, SKA : skeletal abnormalities, SP : skin pigmentation, NA : not available

**Figure 1:**
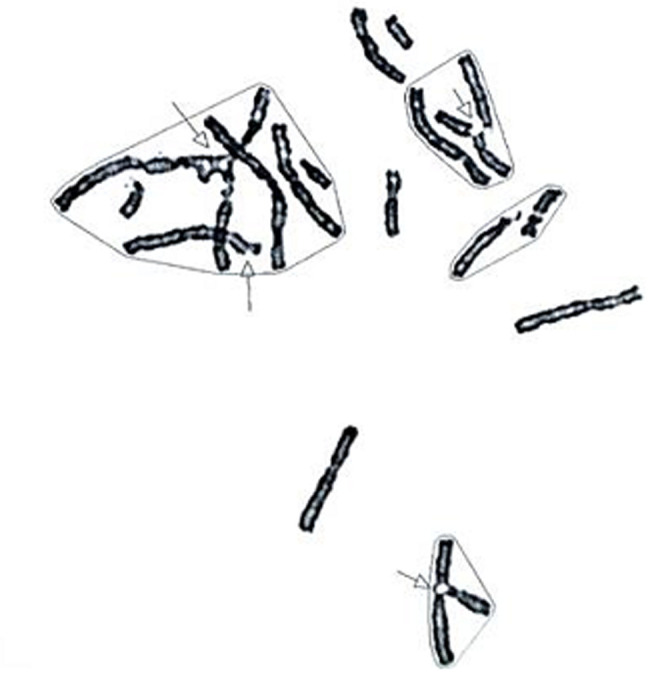
cytogenetic analysis showing chromosomal breaks and radial forms

**Cytogenetic analysis with mitomycin (MMC):** all the patients had a number of chromosomal breaks significantly higher than the normal controls blood with radial forms (Figure 1). A very high instability was observed in three patients (P7, P8, P9) with a number of breaks over than 90 and an increased rate of tri and tetra-radial figures (Table 2).

**Table 2:** cytogenetic results with the number of chromosomal breaks and radial forms compared with the normal control blood

Patients	Chromosomal breaks		Radial forms
	Patient	Control	
P1	36	8	2
P2	64	8	11
P3	34	8	4
P4	21	2	1
P5	39	8	4
P6	83	7	5
P7	92	8	27
P8	128	8	16
P9	170	8	41

**Molecular study:** the molecular analysis shows variations in two of the three genes studied in eight of nine tested patients (88%). The massive sequencing reveals nine variations including four novel sequence changes following autosomal recessive inheritance. All the patients were homozygous except one who was compound heterozygous (Table 3).

**Table 3:** FA gene mutations identified by Next Generation Sequencing among eight Moroccan FA patients

Gene	Patient	Exon	Mutation type	cDNA change	Protein change	Genotype
FANCA	P3	15	Deletion	Exon 15 deletion		homozygous
	P5	13	Nonsense substitution	c.1126C>T	p.Gln376*	homozygous
	P6	36	Non frameshift deletion	c.3520_3522delTGG	p.Trp1174del	Compound heterozygous
		24	Frameshift deletion	c.2189delT	p.Leu730fs	Compound heterozygous
	P8	14	Missense substitution	c.1304G>A	p.Arg435His	homozygous
	P9	29	Missense substitution	c.2851C>T	p.Arg951Trp	homozygous
FANCG	P1	11	Nonsense substitution	c.1474G>T	p.Glu492*	homozygous
	P2	13	Nonsense substitution	c.1642C>T	p.Arg548*	homozygous
	P7	8	Missense substitution	c.1034A>T	p.Gln345Leu	homozygous

***FANCA* gene:** five patients (62.5%) carried FANCA mutations including one homozygous exon 15 deletion and two missense mutations in exon 14 (c.1304G>A, p.Arg435His) and in exon 29 (c.2851C>T, p.Arg951Trp). There was one novel nonsense substitution in exon 13 (c.1126C>T) not found neither in ExAC, and in 1,000 Genomes Browsers. This variation creates a premature translational stop signal at codon 376 in the FANCA protein (p.Gln376*) and expects to result in an absent or disrupted protein product. According to mutationTaster, the protein features might be affected and it is predicted to be damaging based on SIFT algorithm. Only one patient was compound heterozygous, the first variant has been reported as pathogenic in affected individuals with FA (Leiden open-source variation database and clinVar). The c.3520_3522delTGG mutation results in the deletion of one amino acid of the FANCA protein (p.Trp1174del), but preserves the integrity of the reading frame. The second variant is a novel mutation in exon 24 (c.2189delT) which was not found in variations databases. This frameshift deletion leads to the creation of a premature stop signal (p.Leu730Argfs*21) and it is predicted to be probably damaging and damaging on Polyphen2 and SIFT algorithm respectively.

***FANCG* gene:** variations were found in 37.5% of the patients with one known pathogenic mutation in exon 13 (c.1642C>T, p.Arg548*). The two other variations were not previously reported. The first is a missense mutation in exon 8 (c.1034A>T). This sequence change replaces Glutamine with Leucine at codon 345 on the FANG protein (p.Gln345Leu). Based on amino acid prediction software tools, this substitution is probably a disease causing mutation. The second novel variation is located in exon 11, it is a substitution of the Guanine by Thymine on position 1474 (c.1474G>T) leading to the creation of a premature stop codon (p.Glu492*). This variant is not present in population databases and it is expected to be damaging and disease causing on both SIFT algorithm and Mutation Taster.

## Discussion

Fanconi Anemia is considered to be the most common cause of constitutional medullar aplasia. Because of the phenotypic variability observed in FA patients, the diagnosis of FA cannot be based on clinical findings alone. Besides, the diagnosis should not be excluded in the absence of physical abnormalities because they may be present in 60%-75% of individuals. Growth deficiency; abnormal skin pigmentation (e.g., café au lait spots or hypopigmentation); and skeletal system malformations (hypoplastic thumbs) are the most frequent physical features [5]. In our study, 77% of the patients had short stature, followed by skin and skeletal abnormalities present in 44% of the cases. In the first decade of life, patients may exhibit hematological manifestations with abnormal production of blood cells. The anemia can be isolated or associated with the involvement of the other hematopoietic cell line. All of the patients had anemia. A bicytopenia (anemia with thrombocytopenia or leucopenia) was observed in 66%, while pancytopenia was found in 33% of the patients resulting in progressive bone marrow failure. However, none of the patients had clinical evidence of hematological or solid tumors. The cytogenetic analysis in the presence of a clastogen, such as mitomycin C or diepoxybutane, was the first genetic study realized in patients with suspicion of FA [6-8]. This test is positive and confirms the diagnosis in more than 90% of cases. For the remaining cases, the absence of chromosome breaks on peripheral blood cultured lymphocytes can be explained by somatic mosaicism. The karyotype under alkylating agents should be performed on fibroblasts, especially in patients with strong clinical arguments suggestive of FA. All the patient´s cells included in this study showed a significantly increased instability when compared to controls with a higher chromosome breakage rate and radial forms.

The phenotypic variability observed in FA reflects genetic heterogeneity. More than 20 genes have been described with three modes of inheritance (Autosomal recessive, autosomal dominant and X-linked). The products of these FANC genes (-A, B, C, D1 (*BRCA2*)/D2, E, F, G (*XRCC9*), I, J (*BRIP1*), L (*PHF9*), M, N (*PALB2*), O (*RAD51C*), P (*SLX4*), Q (*XPF*), R (*RAD51*), S (*BRCA1*), T (*UBE2T*), U (*XRCC2*), V (*REV7/MAD2L2*), W (*RFWD3*)) interact in a common signalling called FA-BRCA pathway, which is involved in maintaining the integrity of the genome through the control of DNA repair. Disruption of this pathway leads to the common cellular and clinical phenotype observed in FA [9]. Thanks to recent advances in NGS, long genetic analysis is no longer carried out systematically to identify the potential genetic cause of FA. In the last few years, multiple laboratories are offering massively parallel sequencing methodology for FA testing using targeted panels that include all the known FA genes [10]. Other genes known to be associated with other bone marrow failure or chromosome instability disorders can also be included in some panels. Based on the results of the last publications, *FANCA, FANCC* and *FANCG* are the most frequent mutated genes in FA patients representing 60-70%, 14% and 10% respectively [11]. The three genes were included in our panel in order to define the molecular profile in Moroccan patients.

In our study, we could confirm the diagnosis in 8 of the 9 patients tested from unrelated families. Among them, 62.5% carried mutations in *FANCA* gene which is similar to previous findings suggesting that *FANCA* is the first gene involved in FA representing 60 to 70% [11,12]. Until the 18^th^ of January, 2020; 175 variants have been reported as pathogenic mutations in the human genomic variant search engine (VarSome) [13]. The most common type of mutation in the *FANCA* gene is large deletions [11,14]. The deletion of exon 15 was found only in one patient and represents only 20% of the *FANCA* mutations. Moroccan FA patients didn´t have any recurrent mutation contrasting with Tunisian population who have a higher frequency of exon 15 deletion [15]. The *FANCG* gene is the second mutated gene in our patients, it represents 37.5% and it´s relatively higher comparing to 10% reported in several studies [16,17]. Similar to Iranian and Korean populations, no *FANCC* mutation carrier was found in the present study [18,19]. The molecular study is not only important to establish a genetic diagnosis for the proband; but also has a prognosis and therapeutic implications. In addition; the identification of the causal mutation is essential for genetic counselling, prenatal diagnosis and carrier detection for individuals with a family history of FA. Our results support that the NGS approach is an efficient method for detection of the causal mutation in the FA genes. Since the present work was carried out on a limited cohort; other studies including a larger cohort of patients are needed to better characterize the mutational profile of Moroccan patients and to establish genotype-phenotype correlations.

## Conclusion

To the best of our knowledge, this is the first Moroccan study to perform molecular analysis using NGS methods for Moroccan patients with FA. Molecular genetic testing using NGS-based gene panel including FANCA and FANCG genes should be proposed to all Moroccan FA patients diagnosed clinically. Our first results suggest that there are no single specific mutations. Knowing the most frequently mutated genes will allow us to define a molecular diagnostic strategy for FA in Morocco.

### What is known about this topic

- The FA is a clinically and genetically heterogenous disease with more than 20 genes;
- The cytogenetic analysis with alkylants agents confirm the diagnosis especially in patients without a typical phenotype;
- The FANCA, FANCC, and FANCG are the most frequent mutated genes representing 84% of all genes involved in FA.

### What this study adds

- In developing countries, the presence of chromosomal breaks and/or radial forms at the constitutional karyotype with alkylating agents should be offered to all the patients to confirm the diagnosis;
- The molecular analysis of the FANCA and FANCG allowed the diagnosis of 88% of our patients with FA.
